# Understanding acute vertigo in emergency care in a large London teaching hospital: patient and physician perspectives on diagnostic challenges and digital support

**DOI:** 10.1136/bmjopen-2025-108069

**Published:** 2026-01-21

**Authors:** Elvira Cortese, Angus I G Ramsay, Nehzat Koohi, Diego Kaski

**Affiliations:** 1 SENSE Research Unit, Department of Clinical and Movement Neurosciences, Institute of Neurology, University College London, London, UK; 2 Audiology Department, School of Speech-Language Pathology and Audiology, Faculty of Medicine, Universidad de Valparaiso, Valparaíso, Chile; 3 Department of Behavioural Science and Health, University College London, London, UK; 4 Comprehensive Stroke Service, University College London Hospitals NHS Foundation Trust, London, UK

**Keywords:** QUALITATIVE RESEARCH, ACCIDENT & EMERGENCY MEDICINE, Clinical Decision-Making, Neurotology, Stroke medicine

## Abstract

**Abstract:**

**Background:**

Acute vertigo is a common but diagnostically challenging presentation in emergency departments (EDs), where rapid distinction of life-threatening conditions—like stroke—is critical. Patient and clinician perspectives are often overlooked, and real ED needs and possibilities remain poorly understood. While smartphone-based clinical decision support tools (CDSTs) show promise, evidence on required features for trust and adoption is limited. The UK’s 2025 10-Year Health Plan highlights digital innovation and AI in urgent care, underscoring the need to address these gaps.

**Objective:**

To explore the experiences of emergency physicians and patients with acute vertigo during the diagnostic process; identify real-world challenges, needs and opportunities within the ED setting; and assess participants’ perceptions of the acceptability of implementing a smartphone-based decision-support tool (CDST) to aid in acute vertigo diagnosis.

**Design:**

Qualitative study using semi-structured interviews and reflexive thematic analysis.

**Setting:**

Emergency Department of University College London Hospitals NHS Foundation Trust (UCLH), UK.

**Participants:**

10 emergency physicians with experience in managing acute vertigo and 10 patients who had recently presented to the ED with symptoms of acute vertigo.

**Results:**

The analyses identified challenges and needs when diagnosing acute vertigo in the ED and participants’ views on future smartphone-based CDST development to assist the diagnostic process. Clinicians emphasised diagnostic complexity, limited training and system-level constraints—like lack of space, time and resources—as major challenges. Patients emphasised the need for better communication and clearer diagnostic pathways. Both groups saw potential in smartphone-based CDSTs to improve diagnostic efficiency and accuracy by offering structured assessments and helping clinicians identify serious conditions.

**Conclusions:**

This study offers insights into real-world constraints of diagnosing acute vertigo in the ED. Findings suggest that aligning CDST design with clinical workflows, user trust and environmental realities may facilitate adoption and impact in emergency care settings.

**STRENGTHS AND LIMITATIONS OF THIS STUDY**

- Conducted in a large inner-city tertiary hospital, serving a diverse and complex population, which enhances the relevance of the findings.
- This is one of the first qualitative research studies on acute vertigo exploring real-world needs in emergency settings.
- Captures insights from both emergency physicians and patients, providing a more holistic understanding of diagnostic challenges.
- Examines the acceptability of potential diagnostic tools in clinical practice, directly responding to the UK’s 10-year healthcare priorities, emphasising innovation in patients care and supporting the development and implementation of digital diagnostic support tools to improve outcomes.
- The main limitation of this study is its single-centre design.

## Introduction

 Acute vertigo is a common yet diagnostically challenging presentation in the emergency department (ED).^1 2^ While many cases are benign, others, such as stroke, can be life-threatening.^3 4^ Symptoms often overlap, making accurate diagnosis difficult without specialist skills or tools, which are not always available in the ED.^4 5^

Over the past decade, clinical decision rules such as Head Impulse, Nystagmus, Test of Skew, plus hearing test (HINTS Plus) and SponTAneous Nystagmus, Direction, head Impulse test, standiNG test (STANDING) have been developed and validated to support the rapid assessment of acute vertigo and aid in the differential diagnosis of high-risk conditions such as posterior circulation stroke.^46^,^8^ These tools have demonstrated excellent sensitivity and specificity. While their diagnostic performance is well supported by evidence, these tools have not, as yet, been widely embraced by emergency physicians,^9^ and their integration into everyday practice has been relatively limited.^3 4^

At the same time, technological advances, accelerated significantly by the COVID-19 pandemic, have driven the rapid development of mobile diagnostic solutions. Smartphone-based technology, powered by machine learning, is increasingly seen as the future of decision support in emergency care.^10 11^ Mobile tools capable of detecting nystagmus or subtle abnormal eye movements using smartphone cameras or partially automating the HINTS examination are already nearing practical implementation.^12^,^14^

The latest version of the UK’s 10-year healthcare plan reflects a significant shift in government perspective, emphasising the need to accelerate evolution from analogue to digital and to boost the development of diagnostic support tools (DSTs). This strategic direction serves as a mandatory guide to promote and enhance the design, functionality, usability, adoption and scale-up of digital technological clinical decision support tools (CDSTs) that, as smartphone-based apps, may help with the diagnosis of different conditions across the healthcare system.^15^

Nonetheless, there is a marked lack of qualitative evidence capturing the perspectives of both frontline emergency physicians and patients, examining the actual needs of emergency settings, as well as the perceived acceptability and feasibility of integrating such tools into clinical workflows. Such insights are vital to ensure that health innovations are contextually relevant, clinically acceptable and truly effective in complex and high-pressure environments such as EDs.^16^,^18^ In this scenario, new tools may overlook essential real-world constraints or fail to gain acceptability among those they are intended to serve. Acceptability, defined here as the perceived usefulness, ease of use and relevance of the tool to clinical and patient needs, is essential at the early stages of innovation.^18^,^21^

This study explored the diagnostic experience of acute vertigo in EDs from both patient and physician perspectives. To inform future innovation, we examined these key questions: What challenges and needs arise during diagnosis? How acceptable is a smartphone-based decision-support tool? And what factors might support or limit its use in real ED settings? These insights aim to guide the design of practical, user-friendly tools that improve diagnostic accuracy and align with current priorities for digital innovation in urgent care.

## Methods and analysis

### Study design and settings

This qualitative study involved semi-structured interviews with acute vertigo patients and emergency physicians in the ED. The study was conducted at University College London Hospitals NHS Foundation Trust (UCLH), a large urban teaching hospital.

### Participants

Participants were purposively sampled to reflect diverse clinical experience and patient diagnostic histories. Recruitment occurred between April and July 2024. Emergency physicians were eligible if they had at least 5 years of clinical experience and had evaluated patients with acute vertigo. 10 physicians (4 females, 6 males, mean age 41, range 27–56) participated, half of whom had formal training in vertigo assessment, while the remainder reported informal or self-directed training.

Patients were recruited from the UCLH acute vertigo clinic. All had attended the ED within the preceding 6 months due to acute vertigo and had received a confirmed diagnosis or remained undiagnosed. 10 patients participated (4 women, 6 men; mean age 59 years, range 36–80), with diagnoses including vestibular migraine (n=3), unilateral peripheral vestibulopathy (n=3), transient ischaemic attack (TIA) or stroke (n=3), and one with uncertain aetiology. Although no CDST prototype was presented, patients were included to explore their diagnostic experiences and expectations around the potential use of technology in the ED. This approach aimed to capture early perspectives on acceptability, trust and usability to inform user-centred design.

### Sample size and information sufficiency

Following Braun and Clarke’s approach to reflexive thematic analysis,^22^,^25^ data collection and analysis proceeded concurrently and iteratively. Information sufficiency was assessed separately for physicians and patients by monitoring the emergence of new codes and meaningful patterned themes across data. Codes began to recur as early as interviews 2–3, with no new themes identified by interviews 6–7. Notably, the same core themes emerged across both participant groups. The final sample size was determined once the central thematic structure had stabilised, indicating that the data provided sufficient depth and scope for meaningful analysis.

### Data collection

Semi-structured interviews were conducted using open-ended questions (online supplemental appendix 1) that explored participants’ experiences of diagnosis, perceptions of barriers and facilitators and views on potential features and challenges of CDSTs. The guide was iteratively refined following early interviews to enhance clarity and scope. For example, a narrow prompt asking, “What problems did you face in the evaluation process?” was reworded as “How was your experience of being evaluated in the ED for acute vertigo?”.

All interviews were conducted by the lead researcher (EC), with prior qualitative research experience. Interviews took place via Microsoft Teams, lasted 30–40 min (patients) or 20–30 min (clinicians) and were audio-visually recorded with participant consent. Patients participated from home, while clinicians joined from private work settings. Interviews were transcribed verbatim in British English, and field notes were written immediately after each session. A conversational, non-intrusive approach was adopted to encourage open sharing. The study followed Consolidated criteria for Reporting Qualitative research (COREQ) reporting guidelines.

### Analysis

Data were analysed using reflexive thematic analysis within a realist–essentialist paradigm, which focuses on participants’ reported experiences without interpreting underlying meaning. Transcripts were checked for accuracy and uploaded to NVivo12. Coding was conducted inductively by the lead researcher and the co-author (AIGR)—an experienced qualitative researcher—and themes generated through iterative refinement, memo-writing and team discussion.

Once initial themes were developed, they were mapped against the Non-adoption, Abandonment, Scale-up, Spread and Sustainability (NASSS) framework^16 18^ to support interpretation of barriers and facilitators related to technology uptake.

Although not used to generate codes, the NASSS framework helped structure later analysis by connecting participant views to broader implementation domains (eg, technology, adopter, organisation). This allowed us to move from individual experiences to system-level implications for CDST development. Final themes were reviewed and refined in discussion with co-authors to improve coherence and rigour. Supporting quotes were anonymised and labelled (eg, P1 for patients, EP1 for emergency physicians).

### Trustworthiness

The credibility of the findings was enhanced through data triangulation, as key themes consistently emerged across both patient and physician interviews, reflecting diverse yet converging perspectives. Dependability was ensured by maintaining a transparent and systematic research process, with thorough documentation at each stage. Confirmability was supported by grounding all identified themes in multiple direct quotations from participants, alongside an iterative and reflexive analytical approach. Ongoing critical self-reflection was employed to minimise interpretive bias and enhance analytical rigour. To support transferability, rich contextual detail was provided regarding the study setting and participant characteristics, enabling readers to assess the applicability of the findings to other healthcare contexts.

### Patient and public involvement

None.

## Results

The analysis identified four overarching categories and 19 themes describing the barriers, facilitators and contextual factors influencing acute vertigo diagnosis and the perceived value of decision-support tool development in the ED (see online supplemental table ST1).

**Figure 1:**
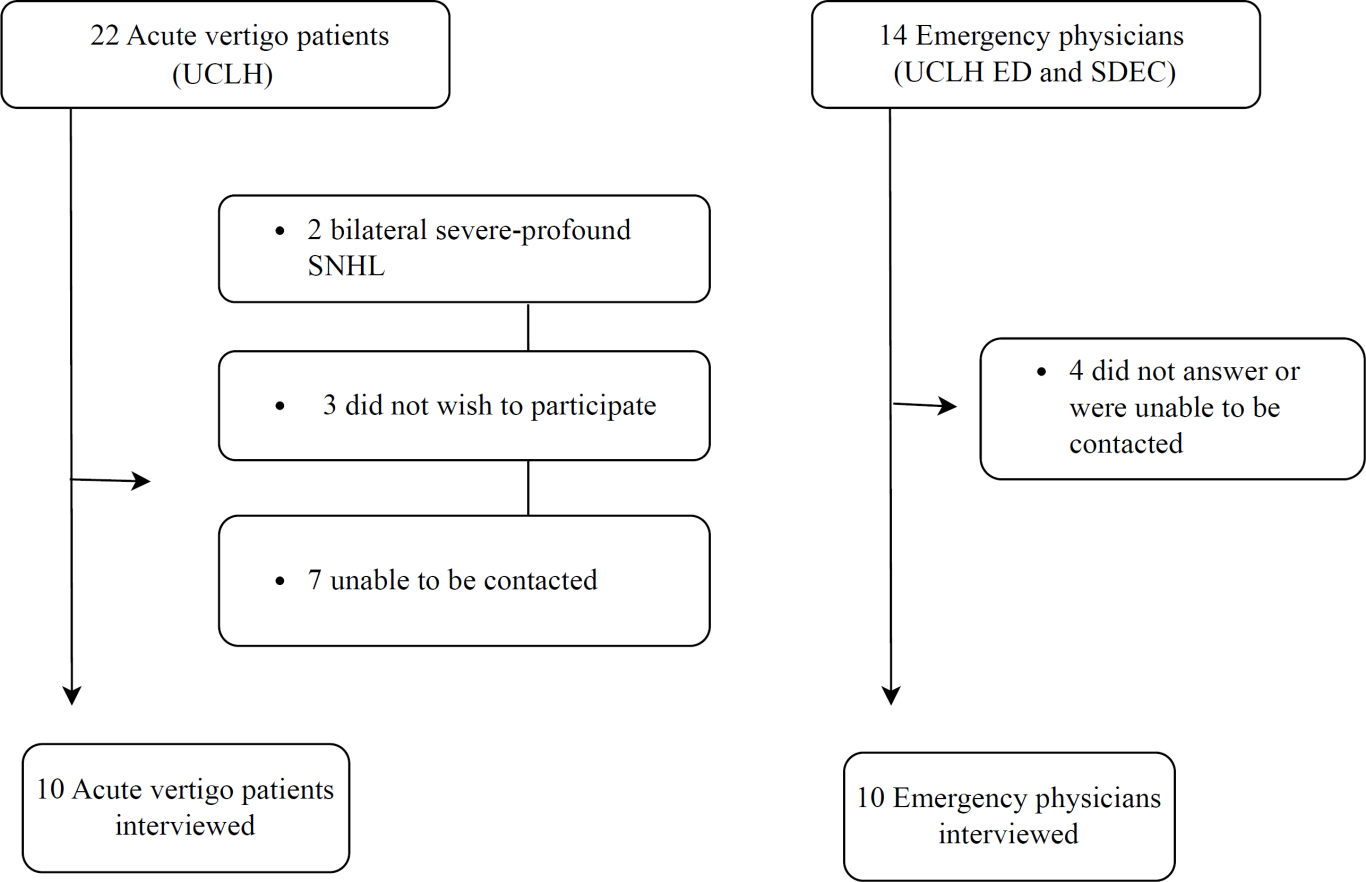
Participants and non-participation. ED, emergency department; SDEC, same day emergency care; SNHL, sensorineural hearing loss; UCLH, University College London Hospitals.

**Figure 2:**
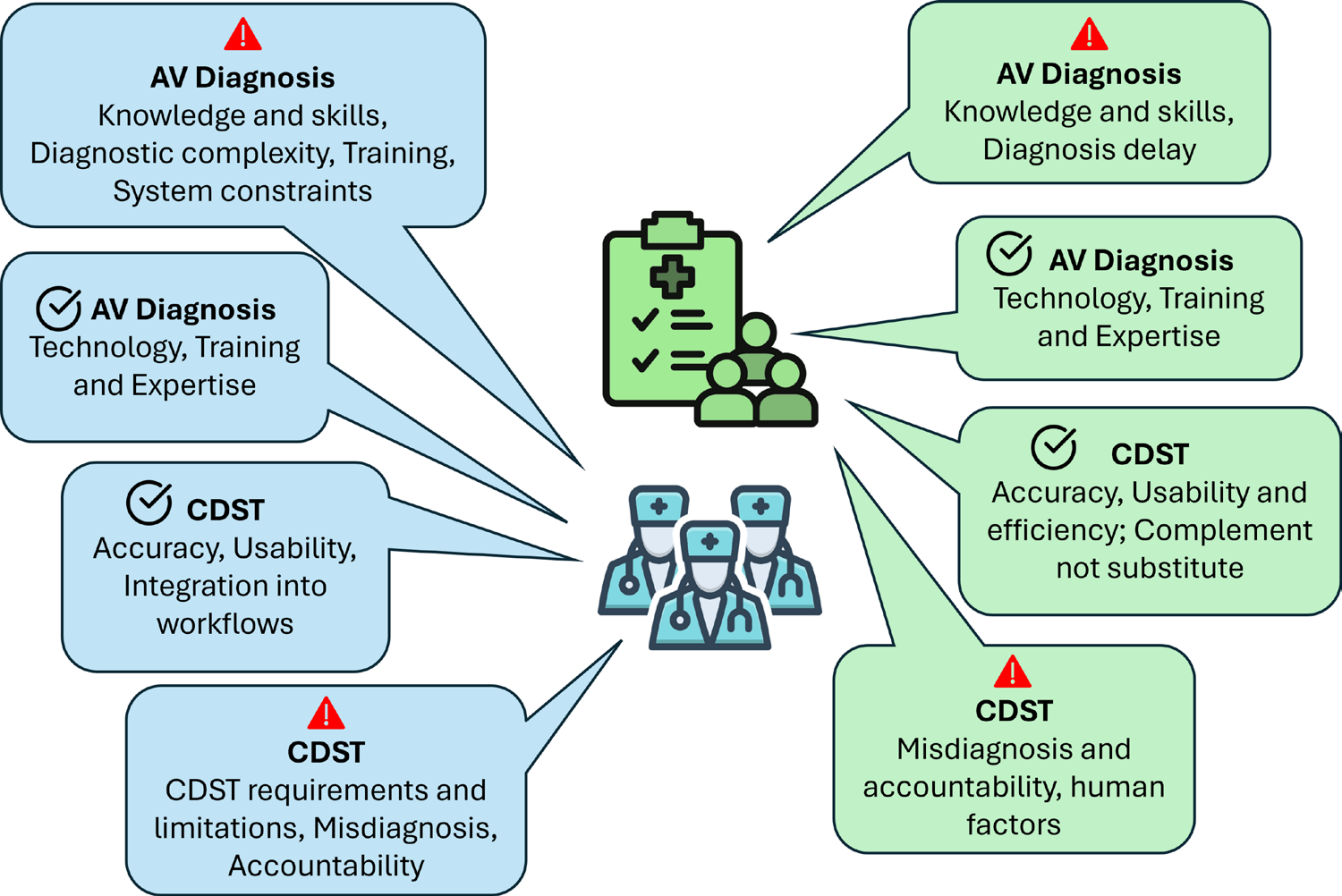
Patients and emergency physicians patterned themes. AV, acute vertigo; CDST, clinical decision support tool; 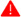 Barriers and 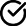 facilitators.

These themes were derived inductively and later mapped onto six domains of the NASSS framework to contextualise findings in relation to healthcare innovation and technology adoption. Figure 1 shows participant flow, and figure 2 offers a visual comparison of the two groups, demonstrating how each theme is represented among clinicians and patients and highlighting the associated facilitators and barriers.

### Barriers for acute vertigo diagnosis in the ED

#### Diagnostic complexity (NASSS: condition)

Emergency physicians often find it difficult to distinguish between peripheral and central causes of vertigo in acutely ill patients due to severe symptoms, unclear histories and time constraints. Patients also report that their intense symptoms hinder communication and make them reluctant to be moved, complicating assessments in the ED.

Sometimes the patient is so symptomatic…very unwell, vomiting etcetera, so, they don't want us to move them at that time in the hyperacute phase… (EPe27: 02EP).

#### Limited training and expertise (NASSS: adopter system)

Several emergency physicians acknowledged limited confidence in acute vertigo assessment and defaulted to treating all cases as potential stroke, increasing reliance on specialist referrals.

We're terrible at diagnosing acute vertigo. 'cause, we think every vertiginous person is a stroke. So, then we ring the stroke team for all of them, and we spend ages trying to work out what’s wrong with them… (EPe6: 07EP).

Patients appreciated their care but felt lost in the ED, sensing that acute vertigo was not well understood and that clinicians lacked expertise in its management.

The staff were very good, I mean, no problems at all with staff or anything like that. Very helpful, very polite, very nice, etcetera, but they just couldn't come up with an answer…but they didn't have an answer… (Pe7: 10AV. Patient)

Nobody seemed to be aware, by the examination, of what my problem was…nobody seemed to really understand how to treat or what to do with vertigo… (Pe6: 03AV. Patient).

#### Diagnosis delay, communication gaps and emotional impact (NASSS: organisation and adopter system)

Patients felt distress over unclear diagnoses and poor communication. An 80-year-old patient noted issues with fragmented care.

I have had an awful lot of tests of all sorts and scans and things, but each time nobody has come up with an answer…to come away from a hospital or an appointment, and nobody knows what’s wrong with you is very frustrating, because you haven't got anywhere. You've still got the problem, but you don't know what it is…And because the point is if you've got something wrong with you, you need to know what it is… (Pe2:10 AV. Patient).

#### System-level constraints (NASSS: adopter system and organisation)

Patients and Physicians described how ED conditions, such as overcrowding and time pressures, hinder vertigo assessment. Physicians often lack adequate environments to perform positional tests or observe subtle eye movements.

If you come to our ED, a lot of these patients end up on chairs rather than trolleys. Doing HINTS or any manoeuvre like that properly on a chair is nearly impossible. (EPe26: 08EP)

Patients highlighted issues in the care pathway, including communication gaps and poor coordination, which forced them to actively seek follow-up and specialist care due to unclear next steps during their ED visit.

The journey was quite difficult …they didn't spend a lot of time explaining, they just gave me a sheet of paper and said: “go home and do that”, So, I don't think is fantastic. Local hospital, I gotta tell you, I don't think they took it that seriously, unfortunately…It wasn't until I actually went back to Professor XX, and she went back to Dr. YY …it’s just dragged on and on and on. I went through my local GP, If I hadn't got the referral to YY, then this wouldn't have escalated anymore… so I had to take it into my own hands… it’s very frustrating… (Pe3: 01AV)

Others noted that even when admitted, little progress was made, leading some to self-discharge out of frustration.

I was there for four days…They weren't doing anything more for me there than what I could do for myself at home…I just, I'd had enough… (Pe5: 08AV)

### Facilitators for accurate diagnosis in the ED

#### Formal training (NASSS: organisation)

Emergency physicians believe improving skills in medical history-taking and physical exams is key to diagnosis, emphasising the need for regular acute vertigo training.

#### Specific technology and expertise (NASSS: value proposition)

Access to structured assessment tools and video-oculography was seen as helpful by both groups. Patients evaluated by a specialist team with access to ocular recordings reported higher satisfaction.

So, she (referring to XX, Specialized Clinical Audiologist) came…she had all of this things… she put me googles, she showed me everything!! (impressed)…It was very reassuring, I knew instantly on that Thursday morning when Dr XX show me the graphs… she just had a laptop, and she could say: “your eyes did this, they didn't do BPPV, they moved the other way”. (Pe18: 02AV. Patient)

Clinicians described specific features that could make technology useful in practice, including the ability to document and analyse eye movements and integrate findings into patient records.

Easy access to diagnostic algorithms and pathways. An easy way to record eye movements in examination findings so that it could be uploaded to patient’s notes or analysed by AI… (EPe7: 01EP).

### Facilitators for decision-support tools in the ED

#### Usability and workflows integration (NASSS: technology)

Acute vertigo patients and emergency physicians stressed the need for user-friendly decision-support tools that integrate into workflows. Tools aiding in history-taking, examination and prioritising serious conditions are especially valuable if they enhance rather than disrupt workflows.

It needs to be easy to use…it needs to be user friendly you know. (Pe28: 08AV)

If it’s something that doesn't need a big space and room to move a patient around, then again, that’s also a good thing (EPe52: 02EP).

#### Acceptability (NASSS: adoption system) and ED efficiency (NASSS: value proposition)

Patients and emergency physicians backed a decision-support tool for diagnosing acute vertigo, seeing it as a means of improving efficiency and reducing delays. While patients sought definitive diagnoses and clinicians prioritised quick triage of severe cases, all agreed on the need for a swift and accurate diagnostic process.

I think anything that could help them, you know, in terms of timing and efficiency, given the pressure they have…, something that could help them to help us. I think would be good… (Pe21: 06AV. Patient).

Would be very helpful for the local doctor or the A&E, to have something that can measure the head and balance between the eye movement versus the head and trying to quickly diagnose, that would be very good, by using something from a smartphone… (Pe22: 01AV. Patient)

I don't think any of us are particularly good at vertigo…so, if we had something automated that would be brilliant… it would be really good to have something quick because it’s not at the moment…I'd like a quick automated thing… (EPe36: 10EP).

To enhance ED efficiency, patients suggested adding multilingual options to overcome language barriers that can hinder communication and crucial clinical history for diagnosing acute vertigo.

Because there’s a lot of nationalities…So, it needs to be able to understand and have…(pause) change of language…that would be really helpful… (Pe39: 08AV. Patient)

It might be able to help with language problems if, you know, if you speak a different language or the doctors speak a different language, that might be helpful… (Pe40: 10AV. Patient)

#### Accuracy

Accuracy was essential for both groups. Clinicians stressed that ED tools must reliably differentiate between benign and serious causes, particularly in time-sensitive situations.

If there is an app or something that will help with the assessment then…it would need to be verified, and accurate, before we'd consider using it as a replacement for what we're doing already… So, it would need to be verifiable. It would need to be reproducible and the results that it obtains would need to be the same or better than what would be obtained in a bedside examination… (EPe43: 02EP)

#### CDST as complement, not a substitute

Clinicians and patients recognised the value of decision-support tools but stressed they should support—not replace—clinical judgement. Patients particularly valued the human aspect of care and were concerned about relying too much on automation in complex cases.

I think it would need to be used in conjunction with doctors…I mean, yes, it might take some of the legwork out of diagnosis to speed it up, but I don't think the human element should be taken away completely, because obviously machines can get it wrong… (Pe35: 10AV)

### Barriers for decision-support tools in the ED

#### Design limitations and special requirements

Clinicians noted that for a decision-support tool to be adopted in the ED, it must integrate seamlessly, as complex systems that increase time pressures are unlikely to be used.

Whatever that is done or whatever is developed, it shouldn't add to our time. It should be something that helps streamline the process… (EPe60: 03EP).

#### Risk of misdiagnosis and accountability

Both groups raised concerns about over-reliance on automated tools.

The generation now it just accepts technology and would probably trust an app… but machines can make mistakes… (Pe49: 10AV).

Clinicians stressed the need for clear delineation of responsibility in the event of diagnostic error.

Medical legal stuff, like if the app gets it wrong, what’s the fault here, you know things like that… (EPe68: 04EP).

#### Organisational constraints

Participants also noted structural challenges such as limited resources, varying digital readiness, lack of training support and resistance to change, as potential barriers to adoption.

#### IT and regulatory challenges

Technical concerns included integration with existing electronic health records, system interoperability and compliance with data protection regulations. These factors were viewed as significant hurdles to scaling up digital tools within National Health Service (NHS) infrastructure.

How much Internet access it needs, cause often you don't get, you're in the ED somewhere and like there’s no signal, and then you're on a slow Wi-Fi and then everything is slow and it doesn't work… (EPe55: 01EP)

#### Human and cultural factors

Patients highlighted human barriers, such as staff pressure and resource shortages, as greater obstacles than material ones. They noted that system complexity and resistance to change could contribute to healthcare staff inertia and indifference.

How a mHealth like this would work on the ED it would depend on how effective the tool is and on how the culture within the particular environment is. So, I mean I did have some experience of a different accident and emergency department recently and it was pretty shocking how poor it was, and how bad the morale was. They didn't seem motivated, or they seem too stressed and overworked … The environment was very challenging because it’s just so crowded, you just had patients on trolleys everywhere, in all the corridors…so I think it would depend probably from hospital to hospital. And it seems to me that a lot of hospitals, well, my limited experience of three hospitals, that some are under incredible stress and strain and aren't functioning properly, incredibly inefficient. So, I wouldn't imagine that (the App use) would work well… (Pe52: 09AV)

## Discussion

This study explored barriers and facilitators to diagnosing causes of acute vertigo in the ED, with a specific interest in the potential role of decision-support tools. The original aim focused on CDST acceptability and development; however, the findings revealed broader issues surrounding the diagnostic process itself, including complexity of presentation, limited clinician training, systemic constraints and patient dissatisfaction.

By incorporating both clinician and patient perspectives, this study provides a validated, experience-grounded understanding of vertigo diagnosis in the emergency setting—an approach not previously described in this context. Such qualitative insights directly informed the design of the decision-support tool, ensuring its features reflect real ED conditions, workflow constraints and patient-centred needs.

While challenges in diagnosing acute vertigo in emergency settings and general concepts surrounding decision-support tools (DSTs) have indeed been discussed in the literature, previous work has largely reflected theoretical or experiential perspectives rather than evidence directly obtained from clinicians and patients in context. While technology may support more accurate and efficient decision making, its success will depend on alignment with clinical workflows, organisational readiness and user acceptability. To our knowledge, this is the first study to empirically confirm these assumptions through the voices of real stakeholders, and importantly, to include patient perspectives alongside those of emergency physicians.

Our findings demonstrated strong support from both patients and emergency physicians for integrating decision-support tools into acute vertigo diagnosis in the ED. However, several implementation challenges must be addressed.^1826^,^28^ Using the NASSS framework, we identified barriers and facilitators across key domains: Condition, Technology, Value Proposition, Adopter System, Organisation and Wider System Context, all of which influence design, adoption and scalability of these tools.

Adhering to implementation science principles, such as the NASSS framework,^16^ may improve DST uptake, reduce non-adoption and ultimately facilitate the scale-up and sustainability of new technologies.

### Understanding the ED context

The first two themes identified in this study related to the current challenges focused on diagnosing acute vertigo, highlighting issues independent of technological innovation. These insights are crucial, as they inform the design of interventions aligned with the ED’s real-world conditions.

Emergency physicians highlighted the difficulty of acute vertigo diagnosis due to gaps in training, diagnostic ambiguity and operational pressure. Both patients and clinicians described how these issues led to misdiagnosis, delays and emotional stress. Patients expect a clear diagnosis; however, the realities of ED care require better communication strategies to manage diagnostic uncertainty. Improvements to referral pathways and differential diagnosis protocols may also help optimise patient care. Ultimately, designing CDSTs that are compatible with the fast-paced and resource-limited ED environment is critical.

### NASSS domain: the condition

Diagnosing acute vertigo in the hyper-acute setting is inherently difficult due to the lack of diagnostic biomarkers and inevitable reliance on clinical history.^29^ Misdiagnoses are frequent, leading to negative health outcomes and significant healthcare costs.^30^,^32^ While EDs prioritise rapid triage for life-threatening conditions, timely and accurate diagnosis remains crucial.

Although EDs prioritise rapid triage of life-threatening conditions,^1 4 33^ this study revealed the importance of timely and accurate diagnosis for improving patient outcomes. Patients emphasised the emotional toll of delayed or incomplete diagnoses, which often necessitate consultations with specialists, weeks or months after their initial ED visit.^34 35^ These diagnostic challenges present a strong case for technological innovation in emergency care.

### NASSS domain: value proposition

CDSTs must deliver tangible value to all stakeholders.^18^ This study shows both patients and emergency physicians perceive value in developing such tools for acute vertigo diagnosis in the ED. Patients want clarity, efficient diagnosis and actionable next steps. These expectations should guide CDST design to ensure clinical relevance, usability and cost-effectiveness^18 28^

### NASSS domain: technology

Given the diagnostic complexity of acute vertigo, ED clinicians could benefit from digital tools to support decision-making. While CDSTs offer promise, they must enhance (rather than replace) clinical reasoning.^16^,^18^ Respondents stressed the need for tools that are accurate, intuitive and work-flow compatible. Smartphone-based technologies were seen as promising if they are reliable, simple and interoperable with existing systems^10^

### NASSS domain: adopter system

Patients highlighted non-technical barriers, such as clinician inertia and resistance to change, which could hinder CDST adoption. The broader healthcare system’s complexity adds further strain. Successful CDST implementation will require engaging both clinicians and patients throughout development to foster ownership and reduce resistance.^10 18 21^

### NASSS domain: organisation and wider system context

Organisational constraints (such as limited resources, training and support) emerged as significant implementation barriers. Furthermore, challenges related to data security, IT compatibility and scalability must be considered. Institutional support and regulatory compliance are essential to ensure sustainable integration of CDSTs into NHS infrastructure and beyond.

### Implications for CDST design and development

This study offers actionable insights for CDST development based on the NASSS framework:

- Address diagnostic complexity through targeted design (the condition).
- Ensure the tool is user-friendly, is minimally disruptive and requires minimal training (technology and adopter system).
- Involve clinicians and patients early in the design process to increase acceptability (adopter system).
- Tailor implementation strategies for ED-specific challenges (organisation)
- Anticipate data, privacy and regulatory needs from the outset (wider system context).

If these principles are adhered to, CDSTs for acute vertigo diagnosis are more likely to be adopted, scaled and sustained^16^,^18^ across NHS EDs and other health systems.

### Strengths and limitations

This study offers valuable and timely insights into the diagnostic challenges and innovation opportunities surrounding acute vertigo in emergency care. We integrated the perspectives of both clinicians and patients, captured the complexity of real-world diagnostic encounters and identified practical barriers and facilitators to guide decision-support tool development. The use of reflexive thematic analysis alongside the NASSS implementation framework strengthens the analytical depth and relevance of the findings, offering direction for technology design that is user-centred and context-aware.

While the study reflects experiences within a specific clinical setting, the themes identified resonate with broader challenges in emergency medicine and digital innovation. As such, these findings may inform efforts to develop, implement and scale diagnostic support across diverse healthcare environments. This study was limited by its single-centre design; however, it was conducted in a large inner-city tertiary hospital, which serves a diverse and complex patient population, enhancing the relevance of the findings. Future research could build on these insights through multi-contextual studies or co-design approaches to further enrich the evidence base for effective and acceptable diagnostic innovation.

## Supplementary material

10.1136/bmjopen-2025-108069 online supplemental file 1

10.1136/bmjopen-2025-108069 online supplemental table 1

## Data Availability

Data are available upon reasonable request.

## References

[1] Edlow JA, Carpenter C, Akhter M (2023). Guidelines for reasonable and appropriate care in the emergency department 3 (GRACE-3): Acute dizziness and vertigo in the emergency department. Acad Emerg Med.

[2] Rau CJ, Terling L, Elkhodair S (2020). Acute vertigo in the emergency department: use of bedside oculomotor examination. *Eur J Emerg Med*.

[3] Herdman D, Ahmad H, Antoniades G (2023). Developing an implementation intervention for managing acute vertigo in the emergency department. Emerg Med J.

[4] Tarnutzer AA, Edlow JA (2023). Bedside Testing in Acute Vestibular Syndrome-Evaluating HINTS Plus and Beyond-A Critical Review. Audiol Res.

[5] Bierrum W, Haider S, Balaratnam M (2023). Hyperacute vestibular syndrome: the role of an acute vertigo service. Front Stroke.

[6] Newman-Toker DE, Kerber KA, Hsieh Y-H (2013). HINTS outperforms ABCD2 to screen for stroke in acute continuous vertigo and dizziness. Acad Emerg Med.

[7] Kattah JC, Talkad AV, Wang DZ (2009). HINTS to diagnose stroke in the acute vestibular syndrome: three-step bedside oculomotor examination more sensitive than early MRI diffusion-weighted imaging. Stroke.

[8] Vanni S, Pecci R, Casati C (2014). STANDING, a four-step bedside algorithm for differential diagnosis of acute vertigo in the Emergency Department. Lo STANDING, un algoritmo bedside a quattro step per la diagnosi differenziale delle vertigini acute nel Dipartimento di Emergenza. ACTA Otorhinolaryngológica Italica.

[9] Warner CL, Bunn L, Koohi N (2022). Clinician’s perspectives in using head impulse-nystagmus-test of skew (HINTS) for acute vestibular syndrome: UK experience. Stroke Vasc Neurol.

[10] Eckhoff RP, Kizakevich PN, Bakalov V (2015). A Platform to Build Mobile Health Apps: The Personal Health Intervention Toolkit (PHIT). JMIR Mhealth Uhealth.

[11] Research 2 Guidance (2013). The app market specialists. Mobile health market report 2013-2017: the commercialization of mHealth applications (vol.3). Vol. 2, JMIR mHealth and uHealth.

[12] Bastani PB, Badihian S, Phillips V (2024). Smartphones versus goggles for video-oculography: current status and future direction. *Res Vestib Sci*.

[13] Friedrich MU, Schneider E, Buerklein M (2023). Smartphone video nystagmography using convolutional neural networks: ConVNG. J Neurol.

[14] Brousseau B, Rose J, Eizenman M (2020). Hybrid Eye-Tracking on a Smartphone with CNN Feature Extraction and an Infrared 3D Model. Sensors (Basel).

[15] UK Department of Health & Social Care, Prime Minister’s Office 10 Downing Street (2025). Fit for the future: 10 year health plan for England-executive summary. https://www.gov.uk/government/publications/10-year-health-plan-for-england-fit-for-the-future/fit-for-the-future-10-year-health-plan-for-england-executive-summary.

[16] Greenhalgh T, Abimbola S (2019). The NASSS Framework - A Synthesis of Multiple Theories of Technology Implementation. Stud Health Technol Inform.

[17] Greenhalgh T, Robert G, Macfarlane F (2004). Diffusion of innovations in service organizations: systematic review and recommendations. Milbank Q.

[18] Greenhalgh T, Wherton J, Papoutsi C (2017). Beyond Adoption: A New Framework for Theorizing and Evaluating Nonadoption, Abandonment, and Challenges to the Scale-Up, Spread, and Sustainability of Health and Care Technologies. J Med Internet Res.

[19] Wisdom JP, Chor KHB, Hoagwood KE (2014). Innovation adoption: a review of theories and constructs. *Adm Policy Ment Health*.

[20] Castle-Clarke S (2017). Falling short: why the NHS is still struggling to make the most of new innovations.

[21] Rt Hon Professor the Lord Darzi of Denham OM KBE FRS FMedSci HonFREng (2024). Independent investigation of the national health service in England. www.gov.uk.

[22] Braun V, Clarke V (2006). Using thematic analysis in psychology. Qual Res Psychol.

[23] Braun V, Clarke V (2023). Toward good practice in thematic analysis: Avoiding common problems and be(com)ing a *knowing* researcher. *Int J Transgend Health*.

[24] Clarke V, Braun V (2013). Successful qualitative research: a practical guide for beginners. https://www.researchgate.net/publication/256089360.

[25] Willig C, Rogers WS, Frosh S (2017). The Sage handbook of qualitative research in psychology.

[26] van Gemert-Pijnen JEWC, Nijland N, van Limburg M (2011). A holistic framework to improve the uptake and impact of eHealth technologies. J Med Internet Res.

[27] Atkins L, Francis J, Islam R (2017). A guide to using the Theoretical Domains Framework of behaviour change to investigate implementation problems. Implement Sci.

[28] Abimbola S, Patel B, Peiris D (2019). The NASSS framework for ex post theorisation of technology-supported change in healthcare: worked example of the TORPEDO programme. BMC Med.

[29] Herdman D (2024). Advances in the diagnosis and management of acute vertigo. J Laryngol Otol.

[30] Newman-Toker DE (2016). Missed stroke in acute vertigo and dizziness: It is time for action, not debate. Ann Neurol.

[31] Kuruvilla A, Bhattacharya P, Rajamani K (2011). Factors associated with misdiagnosis of acute stroke in young adults. J Stroke Cerebrovasc Dis.

[32] Choi JY, Lee SH, Kim JS (2018). Central vertigo. Curr Opin Neurol.

[33] Nham B, Wang C, Reid N (2023). Modern vestibular tests can accurately separate stroke and vestibular neuritis. J Neurol.

[34] Klarendic M, Joffily L, Rodrigues FA (2024). The dizzy patient: duration from symptom onset to specialist review. J Neurol.

[35] Bamiou D-E, Kikidis D, Bibas T (2022). Diagnostic accuracy and usability of the EMBalance decision support system for vestibular disorders in primary care: proof of concept randomised controlled study results. J Neurol.

