# Supplementary material for: Understanding acute vertigo in emergency care in a large London teaching hospital: patient and physician perspectives on diagnostic challenges and digital support
Source: BMJ Open. 2026 Jan 21;16(1):e108069. doi: 10.1136/bmjopen-2025-108069 (PMC12829394; doi:10.1136/bmjopen-2025-108069)
Supplement: online supplemental file 1 [file bmjopen-16-1-s001.docx]

**Appendix 1: Interview Guide**

1. **Questions for the acute vertigo patients**

***Q1. How was the experience of being evaluated in the emergency department when you had acute vertigo?***

The following introduction was used to the following Q2. Question:

“...Acknowledging that at the time you were presenting acute vertigo (dizziness) and feeling very bad, with nausea, maybe vomiting, and possibly with instability. If the technology to improve the diagnosis existed at that time - a smartphone-based app to assist the diagnosis of AV at the emergency department (the diagnostic tool, in simple words, would consider the use of cameras and sensors to capture eye movement, will be audio-guided, and would allow to rule out the causes of acute vertigo in the emergency department) …”

***Q2. What would you think about that (previous introduction to Q2.), we would want to know your ideas about this possibility.***

***Q3. What features do you think this App should have?***

***Q4. What barriers, problems or limitations, if any, do you think could exist for introducing a Smartphone-based decision support tool to assist doctors in the diagnosis of acute vertigo patients in the emergency department?***

1. ***Questions for the frontline emergency physicians at the Emergency Department***

***Q1. How high a priority is an accurate diagnosis of acute vertigo in the emergency department, relative to your other responsibilities and why?***

***Q2. Do you feel the diagnosis of the underlying causes of acute vertigo is timely / quick enough and why?***

***Q3. What sort of support, information or technology would help clinicians like you to make a quicker and more confident diagnosis?***

The following introduction was used to the next Q4. question

“...If the technology to improve the diagnosis existed - a smartphone-based app to assist the diagnosis of acute vertigo at the emergency department (the diagnostic tool, in simple words, would consider the use of cameras and sensors to capture eye movement, will be audio-guided, and would allow to rule out the causes of acute vertigo in the emergency department) …”

***Q4. What would you think about that (previous introduction to the question), we would want to know your ideas about this possibility?***

***Q5. What kind of characteristics or features do you think that support tool should have?***

***Q6. What barriers, problems or limitations, if any, do you think could exist for introducing a Smartphone-based decision support tool to assist doctors in the diagnosis of acute vertigo patients in the emergency department?***
