# Supplementary material for: Understanding acute vertigo in emergency care in a large London teaching hospital: patient and physician perspectives on diagnostic challenges and digital support
Source: BMJ Open. 2026 Jan 21;16(1):e108069. doi: 10.1136/bmjopen-2025-108069 (PMC12829394; doi:10.1136/bmjopen-2025-108069)
Supplement: online supplemental table 1 [file bmjopen-16-1-s002.docx]

| Overarching category | Themes | Aligned NASSS Domains |
| --- | --- | --- |
| Barriers to Acute Vertigo Diagnosis in ED | - Diagnostic complexity | - The condition |
|  | - Limited training and expertise | - The adopter system |
|  | - Diagnosis Delay | - The organisation |
|  | - Communication gaps and emotional impact  - System-level constraints | - The adopter system and the organisation |
| Facilitators for Acute vertigo diagnosis in the ED | - Specific technology and expertise | - The Value proposition |
|  | - Formal training | - The Organisation |
| Facilitators for Decision-Support Tools in ED  (DST must consider) | - Acceptability | - The Adopter System |
|  | - Accuracy  - Usability  - Integration into workflows  - ED Efficiency  - DST as Complement not a substitute | - The Technology |
| Barriers to the Adoption of Decision-Support Tools in ED  (Beware in DST development) | - Special requirements  - Misdiagnosis and accountability  - Design limitations | - The Technology |
|  | - Organisational constraints  - IT and regulatory challenges  - Human and cultural factors | - The Organisation and  - The Wider System Context |

Table 1. Main themes and Alignment with NASSS domains.

Table 1. NASSS: Non adoption, abandonment, scale-up, spread and sustainability framework (Greenhalgh 2017); ED: emergency department; IT: information technology ; CDST: Clinical decision support tool.
