# Supplementary material for: Understanding acute vertigo in emergency care in a large London teaching hospital: patient and physician perspectives on diagnostic challenges and digital support
Source: BMJ Open. 2026 Jan 21;16(1):e108069. doi: 10.1136/bmjopen-2025-108069 (PMC12829394; doi:10.1136/bmjopen-2025-108069)
Supplement: Supplementary file 3 [file bmjopen-2025-108069.reviewer_comments.pdf]

PEER REVIEW HISTORY

BMJ Open publishes all reviews undertaken for accepted manuscripts. Reviewers are asked to complete a checklist review form and are provided with free text boxes to elaborate on their assessment. These free text comments are reproduced below.

ARTICLE DETAILS

Title (Provisional)

Understanding Acute Vertigo in Emergency Care in a large London teaching hospital: Patient and Physician Perspectives on Diagnostic Challenges and Digital Support

Authors

Cortese, Elvira; Ramsay, Angus I. G.; Koohi, Nehzat; Kaski, Diego

---

VERSION 1 - REVIEW

Reviewer 1

Name Mansmann, Ulrich

Affiliation Ludwig-Maximilians-Universitat Munchen, Departement of Medical Information Processing, Biometry, and Epidemiology

Date 24-Sep-2025

COI None

This is a qualitative study based on interviews of 10 clinicians and 10 patients on vertigo experience in the emergency department (ED) in a single-center setting. The focus are real-world challenges and constraints when diagnosing vertigo in ED. It is also of interest how smartphone-based digital decision support tools would fit in a reorganization of respective services. The paper looks at the marked lack of evidence regarding the perspectives of clinicians as well as patients. New tools and strategies should be tailored to the needs of both groups.

The sample size was justified by having no new themes coming up during further interviews. The list of topics was saturated after 7 interviews (in clinicians as well as in patients).

The mechanism of processing the information are well described and look appropriate.

There have been 4 overarching topic categories with in total about 19 themes. Examples of questions and answers provided are given between lines 204 and 370.

A results section is not provided. Results are presented in the discussion section. The discussion gives a short overview on the general tendencies of answered given within the overarching themes. For me the presented content was of now surprise. Especially the

situation on the clinician side is discussed in many papers. Also, the discussion of the patient side did not really provide new and relevant information.

Presenting the patient positions in more detail would have been of high interest for me as an outsider of the ED setting.

A graphical representation of the results would have been very helpful organized by each topic, by patients / clinicians and aspects describing facilitators, barriers.

I do not see new aspects provided in the last section of the discussion (DSTdesign and development). The cited issues are known and are well discussed in the literature without making real progress.

Finally, the COREQ guidelines (Consolidated reporting of qualitative studies) checklist is provided and documents compliance with the guideline.

Reading thorough this manuscript did not open my attention to new issues. It is nice that 20 people could formulate their ideas, but I do not see a real progress which can come up based on the reported (and well known) ideas.

Reviewer **2**

Name **Bruintjes, Tjasse D.**

Affiliation **Leiden University Medical Center, Department of Otorhinolaryngology—Head and Neck Surgery**

Date **12-Oct-2025**

COI **None**

Interesting qualitative research study exploring the factors that both help and hinder implementation of a smartphone-based decision-support tool in acute vertigo diagnosis. The study offers practical insights for development and implementation of such a tool.

This is a new approach to a clinically relevant topic. I am not an expert in qualitative research, but the design of the study seems appropriate. The manuscript is well-written and well-structured.

I have one minor point. For the non-expert it is unclear what a DST (decision-support tool) in acute vertigo diagnosis might be. Only in line 73-74 it becomes clear that is in fact a smartphone detecting eye movements. I would add this information in the abstract.

VERSION 1 - AUTHOR RESPONSE

REVIEWER 1

Dr. Ulrich Mansmann, Ludwig-Maximilians-Universitat Munchen

Comments to the Author:

This is a qualitative study based on interviews of 10 clinicians and 10 patients on vertigo experience in the emergency department (ED) in a single-center setting. The focus are real-world challenges and constraints when diagnosing vertigo in ED. It is also of interest how smartphone-based digital decision support tools would fit in a reorganization of respective services. The paper looks at the marked lack of evidence regarding the perspectives of clinicians as well as patients. New tools and strategies should be tailored to the needs of both groups.

The sample size was justified by having no new themes coming up during further interviews. The list of topics was saturated after 7 interviews (in clinicians as well as in patients).

The mechanism of processing the information are well described and look appropriate.

There have been 4 overarching topic categories with in total about 19 themes. Examples of questions and answers provided are given between lines 204 and 370.

Comments to be answered

1). A results section is not provided. Results are presented in the discussion section.

Response 1:

We would like to respectfully clarify that a Results section was indeed included in our original submission. The Results section begin on page 7 (line 217 onwards) in the main-document-clean now and continue until the Discussion section, which starts on page 12 (line 404 onwards). In both, the first submitted version and in this 'main-document-clean', each section was clearly labelled in capital letters and bold font to distinguish them.

It is therefore possible that a formatting or file-viewing issue during the review process may have prevented the Results section from being displayed or accessed in the version downloaded by the Reviewer.

We would be most grateful if the Reviewer could revisit the Results section in the complete version of the manuscript, as it directly addresses the study findings discussed thereafter.

We sincerely appreciate your careful reading and constructive comments and hope this clarification helps ensure an accurate evaluation of the work.

2). The discussion gives a short overview on the general tendencies of answered given within the overarching themes. For me the presented content was of no surprise. Especially the situation on the clinician side is discussed in many papers. Also, the discussion of the patient side did not really provide new and relevant information.

Response 2: We thank the reviewer for this observation and appreciate the opportunity to clarify the contribution of our discussion section. Our intention was not to claim novelty in each individual theme, but rather to synthesise and critically integrate the perspectives of both clinicians and patients within the same analytical framework — something that, to our knowledge, has not been systematically addressed in previous literature.

While certain aspects of the clinicians' experiences have indeed been discussed in earlier studies, our review highlights the convergence and contrast between clinician and patient perspectives, thereby offering a more comprehensive understanding of shared barriers and facilitators in the diagnostic process of acute vertigo in the emergency department.

Although the challenges surrounding acute vertigo diagnosis in emergency settings have often been mentioned in clinical discussions and publications, this topic largely reflects a widely shared, practice-based understanding rather than evidence grounded in empirical research. To clarify the real state of knowledge, we conducted a systematic review following PRISMA guidelines, consulting five major databases (Web of Science, Medline, Embase, CINAHL, and PubMed).

Out of 322 identified articles, only two met the inclusion criteria and NONE included patient experiences or stakeholder input and thus provided only partial insight.

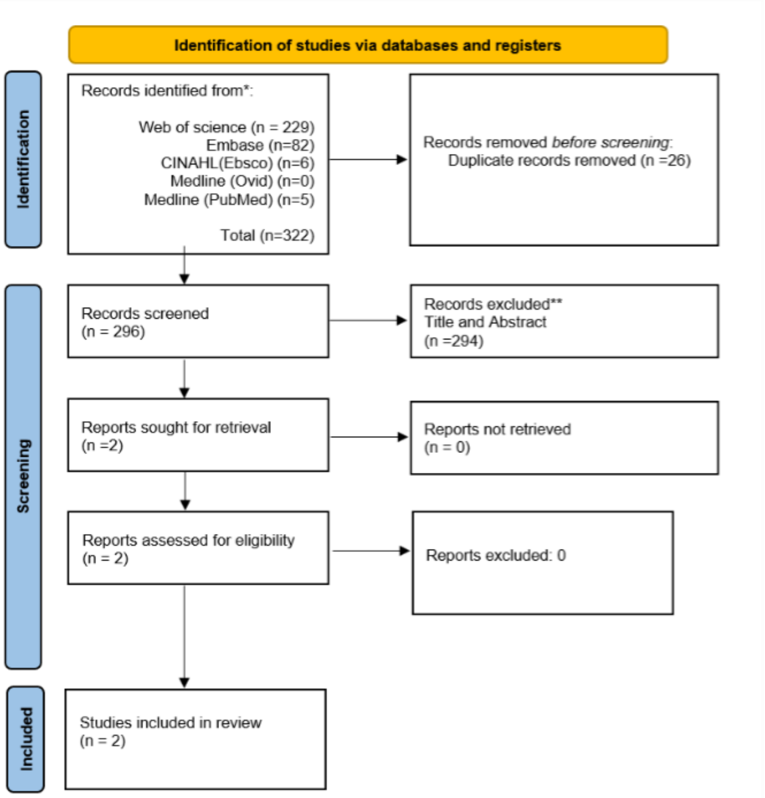

Our qualitative study at UCLH is therefore the first to generate concrete, evidence-based understanding of this issue by integrating the views of both clinicians and patients. It identifies real-world barriers, needs, and opportunities in the diagnostic process and, for the first time ever, evaluates the acceptability of a smartphone-based clinical decision-support tool (CDST) to assist acute vertigo diagnosis in the emergency department

This process confirmed that much of what has been circulated so far constitutes practical or experiential knowledge—widely acknowledged in clinical discussions and practice—but not evidence derived from systematic research involving participants or stakeholders in context.

To strengthen this point, we have now expanded the Discussion section to make these integrative insights more explicit and to better emphasise how our findings contribute to identifying practical implications for the development of supportive diagnostic tools:

> "While challenges in diagnosing acute vertigo in emergency settings and general concepts surrounding decision-support tools (DSTs) have indeed been discussed in the literature, previous work has largely reflected theoretical or experiential perspectives rather than evidence directly obtained from clinicians and patients in context. To our knowledge. This is the first study to empirically confirm these assumptions through the voices of real stakeholders, and importantly, to include patient perspectives alongside those of emergency physicians"

This was added to both; the marked version, page 13 (lines 408 - 416) and in the clean version, page 15 (lines 415 - 422)

3). Presenting the patient positions in more detail would have been of high interest for me as an outsider of the ED setting.

Response 3: We thank the reviewer for this helpful comment. Our study uniquely integrates both clinician **and patient** perspectives to characterise the real-world diagnostic process for acute vertigo in emergency care, revealing a validated and nuanced picture of the diagnostic process, grounded in lived experiences.

The inclusion of patients' voices represents an important advancement: to our knowledge, this is the first study to formally capture and integrate patient perspectives on acute vertigo diagnosis in emergency care. This qualitative foundation directly informed the design of our clinical decision-support tool (DST), translating observed challenges—such as workflow constraints and usability needs—into specific, actionable design criteria.

We believe that this translation of real-world qualitative evidence into concrete design criteria constitutes meaningful progress. Rather than reiterating known problems, the study transforms them into actionable parameters for the design, testing, and implementation of a clinically relevant CDST.

To further address the reviewer's comment regarding patient perspectives, we have expanded this section in the revised manuscript to highlight their unique contributions, specifically informing the CDST design, adding:

> "By incorporating both clinician and patient perspectives, this study provides a validated, experience-grounded understanding of vertigo diagnosis in the emergency setting—an approach not previously described in this context. Such qualitative insights directly informed the design of the decision-support tool, ensuring its features reflect real ED conditions, workflow constraints, and patient-centred needs.
>
> While challenges in diagnosing acute vertigo in emergency settings and general concepts surrounding decision-support tools (DSTs) have indeed been discussed in the literature, previous work has largely reflected theoretical or experiential perspectives rather than evidence directly obtained from clinicians and patients in context. To our

knowledge. This is the first study to empirically confirm these assumptions through the voices of real stakeholders, and importantly, to include patient perspectives alongside those of emergency physicians."

This was added to both; the marked version, page 13 (lines 404 - 416) and in the clean version, page 15 (lines 411 - 422)

…and we removed next paragraph on page 14, integrating part of it into the previous one, and deleting some redundant information.

4). A graphical representation of the results would have been very helpful organized by each topic, by patients / clinicians and aspects describing facilitators, barriers.

Response 4: We thank the reviewer for this suggestion. In the original version, this information was summarised in Table 1, which presents the main themes identified across both groups. However, we fully agree that a graphical representation could make the comparison between patients' and clinicians' perspectives clearer and more accessible.

To address this, we have now prepared a new figure (Figure 2) that visually compares the two groups and illustrates how each theme was reflected among clinicians and patients, including the associated facilitators and barriers. This information was also updated in the 'main document - marked version (Lines 199 – 201)'. We have included it in the marked version, page 8 (lines 223 - 225) and in the clean version, page 7 (lines 231 - 232) and Table 1 was moved to the supplementary materials.

Figure 2:

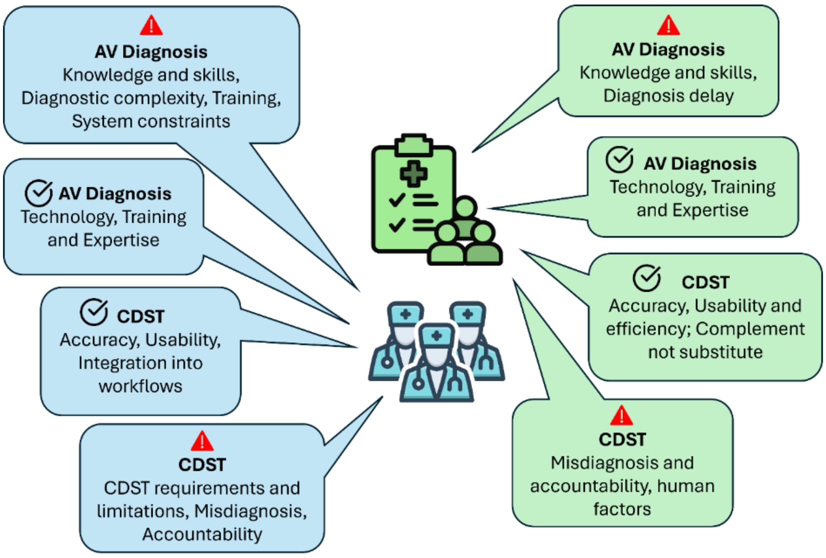

We believe this revision enhances the clarity and visual impact of our results, making the findings easier to interpret while preserving the depth and contextual richness of participants' perspectives.

5). I do not see new aspects provided in the last section of the discussion (DST design and development). The cited issues are known and are well discussed in the literature without making real progress.

Response 5: We thank the reviewer for this observation. We respectfully wish to clarify that the primary aim of this study was to gather, for the first time, real-world empirical evidence to substantiate what has so far been practice-based or informally shared knowledge regarding the diagnosis of acute vertigo in the emergency department.

While the general concepts surrounding decision-support tools (DSTs) have indeed been discussed in the literature, previous work has largely reflected theoretical or experiential perspectives rather than evidence directly obtained from clinicians and patients in context. To our knowledge, this is the first study to empirically confirm these assumptions through the voices of real stakeholders, and importantly, to include patient perspectives alongside those of emergency physicians.

We believe this grounded evidence provides an essential foundation for the design and development of future DSTs, ensuring that such tools are informed by the actual experiences, needs, and barriers encountered in clinical practice

6). Finally, the COREQ guidelines (Consolidated reporting of qualitative studies) checklist is provided and documents compliance with the guideline.

Response 6: We sincerely thank the reviewer for acknowledging this. We made a deliberate effort to adhere closely to the COREQ guidelines throughout every stage of the study, from design to reporting. We believe that following these standards has enhanced the reflexivity, transparency, and methodological rigour of our work, thereby strengthening the credibility and trustworthiness of the findings.

7). Reading thorough this manuscript did not open my attention to new issues. It is nice that 20 people could formulate their ideas, but I do not see a real progress which can come up based on the reported (and well known) ideas.

Response 7: We sincerely thank the reviewer for this feedback and appreciate the opportunity to clarify our study's contribution. We respectfully wish to emphasise that the main objective of this work was not to introduce entirely new conceptual issues, but rather to provide, for the first time, empirical and context-based evidence to substantiate what has so far been informally acknowledged or practice-based knowledge in the field of acute vertigo diagnosis in the emergency department.

To our knowledge, this is the first study to formally document and validate these insights through the direct voices of real stakeholders—clinicians and patients—within the emergency care context. Importantly, this study also represents the first inclusion of patient perspectives on this topic, offering an evidence-based foundation that can inform the future design of supportive diagnostic tools and targeted training strategies.

REVIEWER 2

Prof. Tjasse D. Bruintjes, Leiden University Medical Center, Gelre Hospital

Comments to the Author:

Interesting qualitative research study exploring the factors that both help and hinder implementation of a smartphone-based decision-support tool in acute vertigo diagnosis. The study offers practical insights for development and implementation of such a tool.

This is a new approach to a clinically relevant topic. I am not an expert in qualitative research, but the design of the study seems appropriate. The manuscript is well-written and well-structured.

Comments to be answered

1). I have one minor point. For the non-expert it is unclear what a DST (decision-support tool) in acute vertigo diagnosis might be. Only in line 73-74 it becomes clear that is in fact a smartphone detecting eye movements. I would add this information in the abstract.

Response 1: We are very grateful for your review and observation that help us to improve the clarity of our work. This has been addressed.

The word 'clinical' was added to the 'main document – marked copy' (Abstract: line 10) and 'main document – clean version' (Abstract: line 8).

The idea of the mobile diagnostic solutions as CDST, had been introduced first in 3rd paragraph of page 3 (lines 85 - 90): "technological advances, accelerated significantly by the COVID-19 pandemic, have driven the rapid development of mobile diagnostic solutions. Smartphone-based technology, powered by machine learning, is increasingly seen as the future of decision support in emergency care (10,11) ".

We have also improved next paragraph on page 3 adding text (94 - 97), to better introduce these previous idea:

"This strategic direction serves as a mandatory guide to promote and enhance the design, functionality, usability, adoption, and scale-up of digital technological Clinical Decision Support Tools (CDST) that, as smartphone-based apps, may help with the diagnosis of different conditions across the healthcare system"
